# YouTube as a source of information on familial Mediterranean fever

**DOI:** 10.7717/peerj.20963

**Published:** 2026-03-11

**Authors:** Cem Ozisler, Ayse Bahar Kelesoglu Dincer, Sevinc Can Sandıkcı, Zuhal Ozisler

**Affiliations:** 1 Department of Rheumatology, University of Health Sciences, Ankara Etlik City Hospital, Ankara, Turkey; 2 Department of Rheumatology, Ankara Etlik City Hospital, Ankara, Turkey; 3 Department of Physical Medicine and Rehabilitation, University of Health Sciences, Ankara Bilkent City Hospital, Ankara, Turkey

**Keywords:** Familial Mediterranean fever, Patient education, Quality, Reliability, YouTube

## Abstract

Familial Mediterranean fever (FMF) is the most common hereditary autoinflammatory disease. YouTube is a popular video-sharing platform that both patients and healthcare professionals access for medical information. This study aimed to assess the content, reliability, and quality of the YouTube videos related to FMF. To evaluate video quality and reliability, the Global Quality Scale (GQS) and DISCERN tool were used. Based on GQS scores, videos were categorized into high-, moderate-, and low-quality groups. Four groups were identified in terms of usefulness; useful information, misleading information, useful patient opinion, and misleading patient opinion. The video review was conducted on November 26, 2023. Fifty-three videos that met the inclusion criteria were included in our study. Among these videos, 33 (62.3%) were classified as useful information, 14 (26.4%) as misleading information, two (3.8%) as useful patient opinion, four (7.5%) as misleading patient opinion. Regarding quality, 21 (39.6%) videos were rated as low quality, 13 (24.5%) as moderate quality, and 19 (35.8%) as high quality. Videos uploaded by physicians were significantly more likely to contain useful information (*p* < 0.001), and demonstrated higher GQS and DISCERN scores compared with other groups (*p* = 0.003, *p* < 0.001, respectively). In addition to high-quality videos, there are also lower quality videos on YouTube, which may lead to the spread of misleading information. Therefore, physicians and professional organizations in the field of rheumatology need to make progress in publishing videos according to their quality by collaborating with YouTube and other video sharing sites. Furthermore, encouraging these organizations to share accurate and professionally produced videos on YouTube would benefit healthcare professionals, patients, and their families.

## Introduction

Familial Mediterranean fever (FMF) is the most common hereditary autoinflammatory disease worldwide, characterized by short, recurrent and self-limiting attacks of fever and serositis (Ozdogan & Ugurlu, 2019; Tufan & Lachmann, 2020). FMF is particularly prevalent among Mediterranean ethnic groups such as Turks, Armenians, Jews, and Arabs (Ozdogan & Ugurlu, 2019; Özen, 2018). The prevalence in these populations ranges from 1:500 to 1:1,000, with the highest reported rate of 1:395 in the Central Anatolia region of Turkey (Tufan & Lachmann, 2020; Özen, 2018). Especially in the 20th century, the prevalence of FMF has started to increase all over the world due to migrations from endemic regions to the other countries (Tufan & Lachmann, 2020; Özen, 2018). FMF is an autosomal recessive disease. In 1997, the discovery of the Mediterranean fever (*MEFV*) gene, located on the short arm of the 16th chromosome (16p13.3), was a milestone in elucidating the etiopathogenesis of the disease (Ozdogan & Ugurlu, 2019; Tufan & Lachmann, 2020; Özen, 2018). The *MEFV* gene encodes a 781-amino acid protein called pyrin or marenostrin. Pyrin is an important regulator of inflammation. Mutations of *MEFV* gene cause pyrin dysfunction. As a result of this situation, uncontrolled interleukin (IL)-1 beta production occurs and FMF-related inflammatory attacks are triggered (Ozdogan & Ugurlu, 2019; Tufan & Lachmann, 2020). Colchicine is the mainstay of the therapy in FMF. In cases of colchicine resistance or intolerance, biological therapies must be considered, especially IL-1 antagonists (Ozdogan & Ugurlu, 2019; Tufan & Lachmann, 2020).

In today’s world, the Internet and social media are a part of daily life and video-sharing platforms have become popular sources of information (Drozd, Couvillon & Suarez, 2018; Madathil et al., 2015). Among these platforms, YouTube—launched in 2005 and now hosting over one billion users—receives more than two billion views per day. On average, a new video is uploaded every minute, and each user spends at least 15 min on the site daily (Drozd, Couvillon & Suarez, 2018; Madathil et al., 2015). Recent research has found that eight out of ten Internet users access their health-related information online. In particular, patients with chronic diseases increasingly rely on Internet-based resources to manage their conditions. Approximately 75% of such patients report that their treatment decisions are influenced by information obtained through online health searches (Madathil et al., 2015). Notably, YouTube enables users to upload medical content without any requirement for verification (Guler & Aydın, 2022). In addition, video contents are not revised by an official organization and misleading information can be spread dangerously (Gragnano et al., 2022). Because of this, healthcare providers and government agencies have expressed concern about the accuracy and quality of the information available on this platform (Madathil et al., 2015).

During the writing of our study, there was no article in the literature examining only FMF-related YouTube videos, except for one study that included all autoinflammatory diseases. In that study, a total of 105 YouTube videos were analyzed, of which only 16 were related to FMF (Sasse et al., 2023). Afterwards, another study focusing on FMF and Youtube videos was published (Coşkun et al., 2024). However, the methods and findings of that study differed from ours. Therefore, the present study was designed to analyze the content, reliability, and quality of YouTube videos concerning FMF based on our own data.

## Materials and Methods

YouTube (http://www.youtube.com) was searched using the keyword “familial Mediterranean fever” (FMF) on November 26, 2023. The browser search history was deleted prior to the study to prevent results from being influenced by previous search results. Youtube’s default *“relevance”* mode was used as a filter, as most viewers do. The first 100 videos were included in the study. All videos and their URLs were recorded for analysis, as YouTube search results are subject to change over time. Only English-language videos were included in the study. Non-English, duplicate, irrelevant, advertisement, and audio-free videos were excluded. All videos were independently evaluated and analyzed by two rheumatologists (CO and ABKD), based on the inclusion and exclusion criteria. The Cohen’s kappa coefficient was used to determine inter-rater agreement. In cases of disagreement between CO and ABKD, a third rheumatologist (SCS) reviewed the video, and a consensus was reached.

The following characteristics of each YouTube video were recorded; upload date (to determine the duration on YouTube), video length (in seconds), and the total number of views, likes, and comments. The number of dislikes was also required to calculate the like ratio ([(likes × 100)/(likes + dislikes)]) and the video power index (VPI: [(like ratio × view ratio)/100]) values used to evaluate the video popularity (Sasse et al., 2023). However, in early October 2021, YouTube announced the removal of public dislike counts from the platform. Therefore, the like ratio and VPI values could not be calculated, as the number of dislikes was unavailable. The view ratio for each video was calculated using the following formula: [views/day] (Sasse et al., 2023).

Videos were categorized into four groups based on the upload source: physician, patient, health related websites, and other (non-medical independent user, non-medical media organization).

They were also classified according to their target audience as healthcare professionals, patients, and non-classified.

All included videos were evaluated for usefulness and classified into four mutually exclusive categories (Tolu et al., 2018):

1. **Useful information:** These videos contained medically useful and accurate information for learning about FMF.
2. **Misleading information:** These videos contained clinically incorrect or unproven information based on currently available scientific evidence. If the video contained partially useful and partially misleading information, it was classified as misleading.
3. **Useful patient opinion:** These videos contained a patient’s personal experience and/or feelings that were useful with regards to the FMF.
4. **Misleading patient opinion:** These videos contained a patient’s personal experience and/or feelings which were not useful or with misleading opinions with regards to the FMF.

The quality of each video was assessed using the Global Quality scale (GQS), which was also used in previous similar studies (Sasse et al., 2023; Coşkun et al., 2024; Tolu et al., 2018; Bernard et al., 2007). The GQS is a five-point scale, with higher scores indicating better video quality. Scores of 4–5 points were considered high quality, 3 points moderate quality, 1–2 points low quality.

Video reliability was evaluated using the modified DISCERN tool, which has also been used in several similar studies (Sasse et al., 2023; Tolu et al., 2018; Singh, Singh & Singh, 2012). Each question is answered as “yes” (scored 1 point) or “no” (scored 0 points), with total scores ranging from 0 to 5. Higher scores represent greater reliability. The questions of the GQS and modified DISCERN tool are presented in Table 1.

**Table 1:** Assessment tools for reliability and quality.

**Modified DISCERN toll** (1 point for every “Yes,” 0 points for “No”)
1. Are the aims clear and achieved?
2. Are reliable sources of information used? (i.e., publication cited, speaker is rheumatologist)
3. Is the information presented balanced and unbiased?
4. Are additional sources of information listed for patient reference?
5. Are areas of uncertainty mentioned?
**Global Quality Scale (GQS)** (Select the appropriate one)
1. Poor quality, poor flow of the site, most information missing, not at all useful for patients.
2. Generally poor quality and poor flow, some information listed but many important topics missing, of very limited use to patients.
3. Moderate quality, suboptimal flow, some important information adequately discussed but others poorly discussed, somewhat useful for patients.
4. Good quality and generally good flow, most of the relevant information listed but some topics not covered, useful for patients.
5 Excellent quality and excellent flow, very useful for patients.

### Statistical analysis

All the data were analyzed statistically using the Statistical Package for the Social Sciences (SPSS) version 22 (SPSS Inc., Chicago, IL, USA). Prior to analysis, the Shapiro–Wilk test was used to assess the normality of data distribution. Non-normally distributed continuous variables were expressed as median (minimum–maximum) values, while categorical variables were expressed as counts and percentages. The Kruskal-Wallis test was used to detect statistically significant differences among more than two independent groups. In cases where a significant difference was identifie in the Kruskal-Wallis test, pairwase comparisons were evaluated using the Mann–Whitney U Test with Bonferroni correction to determine the groups that led to significant differences. The *χ*^2^ test was used to evaluate categorical variables. The relationship between variables were analyzed using Spearman’s correlation coefficients. The inter-rater agreement was assessed using the Cohen’s kappa coefficient. A *p* value of < 0.05 was considered statistically significant.

## Results

A total of 100 videos were initially screened. Of these, six duplicate videos, 19 videos presented in languages other than English (nine in Turkish, six in Arabic, two in German, and two in French), 17 irrelevant videos, three advertisement videos, and two videos without audio were excluded from the study. Then, 53 videos that met the determined criteria were selected for further analysis. The features, usefulness categories, quality stratification, upload sources, and target audiences of these videos are summarized in Table 2.

**Table 2:** Baseline characteristics, usefulness categories, and quality stratification of the analyzed videos.

Video features	Median (minimum–maximum)
Number of days on YouTube	1,960 (131–4,662)
Duration in seconds	225 (33–3,090)
Number of views	761 (5–32,133)
View ratio	0.45 (0.02–20.09)
Number of likes	10 (0–461)
Number of comments	0 (0–156)
GQS score	3 (1–5)
DISCERN score	3 (1–5)
**Usefulness categories of the videos**	**n (%)**
Useful information	33 (62.3)
Misleading information	14 (26.4)
Useful patient opinion	2 (3.8)
Misleading patient opinion	4 (7.5)
**Quality stratification of the videos**	**n (%)**
Low quality	21 (39.6)
Moderate quality	13 (24.5)
High quality	19 (35.8)
**Source of upload**	**n (%)**
Physician	27 (50.9)
Patient	6 (11.3)
Health related websites	13 (24.5)
Other	7 (13.2)
**Target audience**	**n (%)**
Patients	24 (45.3)
Healthcare professionals	21 (39.6)
Non-classified	8 (15.1)

The videos were categorized into three groups based on their GQS scores. Except for the number of days on YouTube, all other video features were significantly higher in the high-quality group compared with the moderate- and/or low-quality groups. The moderate-quality group had significantly higher scores than the low-quality group only in terms of the DISCERN score. To determine which groups the statistical differences originated from, pairwise comparisons were conducted using the Mann–Whitney U test with Bonferroni correction, and a *p*-value of < 0.017 was considered statistically significant. When a significant difference was detected, the corresponding letter was added to the upper right of the *p*-value (Table 3).

**Table 3:** Comparison of the video features according to the their quality stratification.

Video features	High quality *n* = 19 (35.8%)	Moderate quality *n* = 13 (24.5%)	Low quality *n* = 21 (39.6%)	p^*^ value
Number of days on YouTube	1,253 (131–4,650)	2,155 (643–4,660)	2,240 (242–4,662)	0.271
Duration	493 (112–3,028)	205 (96–1,557)	181 (33–3,090)	***0.011*** ^b^
Number of views	1,666 (39–32,133)	1,504 (99–21,150)	303 (5–4,118)	***0.01*** ^a,b^
View ratio	1.33 (0.06–20.09)	0.64 (0.13–5.55)	0.12 (0.02–1.82)	***<0.001*** ^a,b^
Number of likes	20 (0–461)	12 (0–230)	2 (0–36)	***<0.001*** ^a,b^
Number of comments	2 (0–66)	1 (0–156)	0 (0–12)	***0.004*** ^b^
DISCERN score	4 (3–5)	3 (2–4)	1 (1–2)	***<0.001*** ^a,b,c^

**Notes.**

All data were expressed as median (minimum-maximum).

* Kruskal–Wallis test, *p* < 0.05 was considered significant and marked bold in the table.

a Statistical evaluation of the video feature between high and moderate quality groups.

b Statistical evaluation of the video feature between high and low quality groups.

c Statistical evaluation of the video feature between moderate and low quality groups.

a, b, and c were determined as a result of the Mann–Whitney U test performed with Bonferroni correction between the paired groups specified in their definitions. Due to Bonferroni correction, a *p* value of < 0.017 between paired groups was considered significant. If there was significance, the appropriate letter was added to the upper right part of the *p* value.

When the videos were analyzed in four groups based on their usefulness, all video characteristics—except for the number of days on YouTube—showed statistically significant differences among the groups. To determine which groups contributed to these differences, pairwise comparisons were performed using the Mann–Whitney U test with Bonferroni correction, and a *p*-value of < 0.0125 was considered statistically significant. When a significant difference was observed, the corresponding letter was added to the upper right of the *p*-value. The findings are summarized in Table 4.

No statistically significant differences were observed between the useful information and useful patient opinion groups in terms of main variables (*p* > 0.05, Table 4). However, the median values of number of views, likes and comments were numerically higher in the useful patient opinion group. Videos uploaded by physicians were found to be significantly more useful than those uploaded by other sources (patients, health related websites, and other) (Table 4).

In terms of upload source, GQS scores were found to be significantly higher in the physician group than in the patient, health-related websites, and other groups [4 (2–5), 1.5 (1–3), 3 (1–4), and 1 (1–4), respectively; *p* = 0.003]. The same statistical evaluation was determined for DISCERN scores [3 (2–5), 1.5 (1–3), 2 (1–5), and 1 (1–2), respectively; *p* <0.001]. Additionally, video durations were significantly longer in the physican group [479 (107–3028), 231.5 (199–3090), 147 (33–733), and 148 (39–2685), respectively].

**Table 4:** Analyses of video characteristics by categories of usefulness.

Video features median (min–max)	Useful information	Misleading information	Useful patient opinion	Misleading patient opinion	p^*^ value
Number of days on YouTube	1,350 (131–4,660)	2,829 (242–4,662)	2,225.5 (643–3,808)	835.5 (451–2,268)	0.143
Duration	381 (96–3,028)	147 (33–779)	231.5 (219–244)	1,605.5 (199–3,090)	**0.012** ^a,e^
Number of views	1,324 (39–32,133)	323.5 (5–2,758)	12,010.5 (2,871–21,150)	304.5 (26–4,118)	**0.005** ^a^
View ratio	0.7 (0.1–20.1)	0.1 (0.02–0.59)	5 (4.5–5.6)	0.6 (0.03–1.82)	**<0.001** ^a^
Number of likes	14 (0–461)	1.5 (0–25)	156.5 (83–230)	17 (3–36)	**<0.001** ^a^
Number of comments	1 (0–66)	0	78 (0–156)	5.5 (0–12)	**0.002** ^a,d,e^
GQS score	4 (1–5)	1 (1–2)	3 (3–3)	1 (1–2)	**<0.001** ^a,c,d,f^
DISCERN score	3 (1–5)	1 (1–2)	2.5 (2–3)	1 (1–2)	**<0.001** ^a,c,d^
***Source of upload, n (%)***					p^**^ ** value**
Physician	24 (72.7)	3 (21.4)	0 (0)	0 (0)	**<0.001**
Patient	0 (0)	0 (0)	2 (100)	4 (100)	
Health related websites	8 (24.2)	5 (35.7)	0 (0)	0 (0)	
Other	1 (3)	6 (42.9)	0 (0)	0 (0)	

**Notes.**

* Kruskal–Wallis test.

** *χ*^2^ test. *p* < 0.05 was considered significant and marked bold in the table.

a Statistical evaluation of the video feature between useful and misleading information groups.

b Statistical evaluation of the video feature between useful information and useful patient opinion groups.

c Statistical evaluation of the video feature between useful information and misleading patient opinion groups.

d Statistical evaluation of the video feature between misleading information and useful patient opinion groups.

e Statistical evaluation of the video feature between misleading information and misleading patient opinion groups.

f Statistical evaluation of the video feature between useful and misleading patient opinion groups.

a, b, c, d, e and,f were determined as a result of the Mann Whitney U test performed with Bonferroni correction between the paired groups specified in their definitions. Due to Bonferroni correction, a *p* value of < 0.0125 between paired groups was considered significant. If there was significance, the appropriate letter was added to the upper right part of the *p* value.

A significant moderate positive correlation was detected between GQS and DISCERN scores. Except for the number of days on YouTube, significant positive correlations were found between other video features (video duration, number of views, view ratio, number of likes, and comments) and GQS/DISCERN scores (Table 5).

**Table 5:** Analysis of the correlation between GQS, DISCERN and video features.

**Video features**	**GQS**		**DISCERN**	
	*r*	*p* ^*^	*r*	*p* ^*^
Number of days on YouTube	−0.182	0.192	−0.148	0.290
Duration in seconds	0.401	**0.003**	0.393	**0.004**
Number of views	0.472	**<0.001**	0.525	**<0.001**
View ratio	0.627	**<0.001**	0.659	**<0.001**
Number of likes	0.550	**<0.001**	0.627	**<0.001**
Number of comments	0.454	**0.001**	0.544	**<0.001**
GQS	–	–	0.901	**<0.001**
DISCERN	0.901	**<0.001**	–	–

**Notes.**

* Spearman’s correlation coefficients, values of *p* < 0.05 were considered as significant and marked bold in the table.

GQS, Global Quality Scale.

Cohen’s kappa coefficient for inter-rater agreement was 0.829 (*p* < 0.001) for the GQS score, 0.848 (*p* < 0.001) for the DISCERN score, and 1.000 (*p* < 0.001) for video usefulness.

## Discussion

Today, the internet is widely used, and many people turn to video-sharing platforms such as YouTube to obtain information on various topics, particularly health-related issues. However, the diversity of authorship and the absence of peer-review process on this platform have led to the spread of false and misleading information (Delli et al., 2016; Kunze, 2020) because any YouTube user, regardless of their health knowledge, experience, or professionalism, can upload a video to this platform. For this reason, YouTube videos related to rheumatological diseases have been evaluated in terms of their usefulness, reliability, quality and accuracy. These studies have shown that, there are videos containing useful and quality information, as well as videos containing missing and incorrect information (Delli et al., 2016; Ng, Lim & Fong, 2020; Onder & Zengin, 2021; Onder & Zengin, 2023; Elangovan, Kwan & Fong, 2021).

FMF is a rare disease, and it can be difficult to reach specialists on this condition. For this reason, patients and individuals seeking information about FMF often turn to the internet, particularly to video-sharing platforms. The findings of the two studies conducted to date have shown differing results regarding the evaluation of FMF-related videos (Sasse et al., 2023; Coşkun et al., 2024). Therefore, we aimed to present and compare our own data.

In our study, the majority of non-English videos were in Turkish and Arabic. This finding can be explained by the widespread use of these languages in geographical regions where FMF is prevalent (Ozdogan & Ugurlu, 2019; Tufan & Lachmann, 2020; Özen, 2018).

The majority of the videos were uploaded by physicians (50.9%), followed by health related websites, other sources and patient-sourced videos (24.5%, 13.2%, 11.3%, respectively). The primary target audience of the videos was patients (45.3%), followed by healthcare professionals (39.6%) and non-classified audience (15.1%). This distribution is consistent with findings from previous studies (Sasse et al., 2023; Coşkun et al., 2024; Ng, Lim & Fong, 2020; Onder & Zengin, 2021; Onder & Zengin, 2023; Elangovan, Kwan & Fong, 2021; Pamukcu & Izci Duran, 2021; Koo, Kim & Jun, 2021). These data give the impression that most of the sources are reliable. Considering the target audience, it is thought that it may be suitable for informational use by patients or their relatives and for educational purposes by healthcare professionals. However, whether these videos are indeed suitable for such purposes should be further evaluated in conjunction with the other findings of our study.

When the videos were evaluated collectively, the median GQS score was 3 (1–5). Previous studies have reported similar (Ng, Lim & Fong, 2020; Pamukcu & Izci Duran, 2021; Zengin & Onder, 2021), lower (Sasse et al., 2023), or higher (Coşkun et al., 2024; Onder & Zengin, 2021; Onder & Zengin, 2023) values. In one study assessing YouTube videos on autoinflammatory diseases, the GQS score for the FMF group was as low as 2 (1–5) (Sasse et al., 2023), whereas another study focusing solely on FMF reported a considerably higher score of 5 (1–5) (Coşkun et al., 2024). The first study included only 16 FMF-related videos, which may explain the low average score. Furthermore, these differences may be attributable to factors such as the inclusion criteria, timing of the search, and variability among raters in both studies. Although this finding clearly indicates a discrepancy, we believe that the GQS distribution observed in our study is more accurate and representative. One study reported a very low GQS value, while another reported a very high value. The GQS value obtained in our study lies between these two extremes and is therefore considered more consistent and compatible with the existing literature.

According to GQS scores, videos were classified as high (35.8%), moderate (24.5%), and low quality (39.6%). While these proportions are consistent with some prior studies (Zengin & Onder, 2021; Kocyigit, Akaltun & Sahin, 2020), others reported different distributions (Onder & Zengin, 2021; Chang, Lee & Park, 2022; Kocyigit & Akyol, 2021). In another study examining FMF-related YouTube videos (Coşkun et al., 2024), the proportion of high-quality videos was remarkably high (72.9%), whereas medium- and low-quality videos accounted for 16.9% and 10.2%, respectively. Although the videos analyzed in both studies were related to the same disease, this article showed the least similarity to our findings among the studies available in the literature. Although this raises the possibility that different videos were analyzed due to variations in evaluation methods or search keywords, the fact that the proportions differ so greatly remains quite thought-provoking.

When GQS groups were evaluated in terms of DISCERN scores; the high-quality group had significantly higher DISCERN scores than both the intermediate- and low-quality groups. Similarly, the intermediate-quality group had higher DISCERN scores than the low-quality group. This finding is consistent with previous studies (Coşkun et al., 2024; Pamukcu & Izci Duran, 2021; Zengin & Onder, 2021). In addition, we found a significant positive correlation between GQS and DISCERN scores, indicating that as the quality of the videos increases, their reliability also increases.

The number of views, view ratio, and number of likes were also significantly higher in the high-quality group compared to the intermediate- and low-quality groups. These higher values may suggest that the quality of a video is better. When evaluated in terms of video duration and number of comments, the high-quality group also showed higher numerical values than both the intermediate- and low-quality groups. However, statistical significance was observed only between the high- and low-quality groups. Previous studies have reported both similar and different findings (Coşkun et al., 2024; Pamukcu & Izci Duran, 2021; Zengin & Onder, 2021; Kocyigit, Akaltun & Sahin, 2020; Kocyigit & Akyol, 2021). Based on this information, if a video is longer and has a higher number of comments, it may indicate better quality. Of course, the higher numerical values discussed in this paragraph do not necessarily guarantee that video quality will always be high.

As of early October 2021, YouTube stopped publicly sharing the number of dislikes, so the like ratio and VPI values could not be calculated in our study. However, in a previous study related to FMF and YouTube videos (Coşkun et al., 2024), the number of dislikes was assumed to be zero for all evaluated videos. Accordingly, in the formula used to calculate the like ratio and VPI values, the dislike value was set to zero. However, we believe that performing such an evaluation without access to the actual dislike numbers may lead to inaccurate results. For instance, in the aforementioned study, no statistically significant difference was found between the low-, medium-, and high-quality groups in terms of either like ratio or VPI values. Nevertheless, if these evaluations had been conducted using the real dislike data, the results might have been different. Therefore, we consider that the discussion based on these data is open to interpretation and that its validity should be questioned.

The median values for the number of views, likes, and comments were numerically higher in the useful patient opinion group, although the difference was not statistically significant. There were studies in which this situation was the same (Tolu et al., 2018) and similar (Ng, Lim & Fong, 2020; Kocyigit & Akyol, 2021). Three possible explanations may account for this observation. First, patients often seek to hear about the experiences of others with the same disease and to identify similarities and differences with their own situation. Second, videos created by healthcare professionals may be less appealing to patients because they often contain excessive medical information. Finally, videos with a high number of views may also have more likes and comments.

GQS and DISCERN scores were significantly higher in the physician group compared to other video sources (patient, health-related websites, and other). Similar findings have been reported in previous studies (Tolu et al., 2018; Onder & Zengin, 2021; Onder & Zengin, 2023; Elangovan, Kwan & Fong, 2021). Although this finding is not surprising, it is noteworthy that it was confirmed through scientific comparison. Based on this evidence, physician-sourced videos can be recommended to individuals seeking information about FMF on YouTube. We recommend that authorities and YouTube administrators take initiatives to increase the number of videos produced by physicians. Moreover, highlighting these videos in search results, or even marking them as “recommended”, could help disseminate accurate and high-quality information.

A significant positive correlation was observed between GQS and DISCERN scores, consistent with some previous studies (Pamukcu & Izci Duran, 2021; Chang, Lee & Park, 2022). Based on this, it can be interpreted that videos with high quality scores may be more reliable. The DISCERN scoring system evaluates whether the information and sources used are reliable, balanced, and free from bias; therefore, high DISCERN scores can serve as an indicator of high quality.

Except for number of days on YouTube, significant positive correlations were found between other video features (video duration, number of views, view ratio, number of likes, and number of comments) and GQS/DISCERN scores. Although no previous study has correlated all these parameters simultaneously with GQS and DISCERN scores, some studies reported similar findings (Coşkun et al., 2024; Pamukcu & Izci Duran, 2021; Chang, Lee & Park, 2022).

Longer videos tended to have higher GQS and DISCERN scores, which can be interpreted as longer videos providing more comprehensive information. We found that videos in the physician group were longer and had higher GQS and DISCERN scores than those in the other groups. Based on these findings, physician-uploaded videos can be recommended as a first choice. Additionally, higher numbers of views, likes, and comments may also serve as useful indicators when selecting videos.

## Limitations

Our study has several limitations, similar to previous YouTube studies. First, it is a cross-sectional study, evaluating only a snapshot at a specific point in time. YouTube is a dynamic video-sharing platform, with content that is constantly changing and expanding. Second, video evaluations are inherently subjective, although a sufficient Kappa score was achieved to indicate inter-rater agreement. Third, only English-language videos were included. Fourth, because YouTube no longer shares dislike counts, like ratio and VPI values could not be calculated in this study. Finally, in our study, only YouTube videos were evaluated, videos on other social media platforms were not analyzed.

## Conclusion

In addition to high-quality videos, most of which are uploaded by physicians, there are also lower-quality videos on YouTube, which may lead to the spread of misleading information. Enhancing the accuracy and reliability of online health information, and having it monitored by health authorities when necessary, would benefit many patients—particularly those with limited access to healthcare—and individuals seeking health information. For this reason, physicians and professional organizations in the field of rheumatology need to make progress in publishing videos according to their quality by collaborating with YouTube and other video sharing sites. Additionally, these organizations could share reliable videos targeting healthcare professionals, patients, and their families. We believe that it would be an important step for health authorities to implement programs guiding the public in accessing accurate information, in addition to digital health education programs for patients.

## Supplemental Information

10.7717/peerj.20963/supp-1 Supplemental Information 1 Raw data

## Additional Information and Declarations

### Data Availability

The following information was supplied regarding data availability: The data is available in the Supplemental File and at YouTube: 1. https://www.youtube.com/watch?v=G7yiB4oEYqs
2. https://www.youtube.com/watch?v=OsZkXgROGpE
3. https://www.youtube.com/watch?v=KFeqZPPFqvg
4. https://www.youtube.com/watch?v=I6ceZq2ABC0
5. https://www.youtube.com/watch?v=X3bibRaHXGw
6. https://www.youtube.com/watch?v=ZMO0915kUm4
7. https://www.youtube.com/watch?v=s3HpJWPOfXg
8. https://www.youtube.com/watch?v=tHVfmfRbhSk
9. https://www.youtube.com/watch?v=ILRjHme7pDw (link not public at time of publication) 10. https://www.youtube.com/watch?v=hJIUt0dQDgA
11. https://www.youtube.com/watch?v=_zTOdVRgiAM
12. https://www.youtube.com/watch?v=Cu9Lk8r-2HA
13. https://www.youtube.com/watch?v=2DBWqG2dt2Y
14. https://www.youtube.com/watch?v=G9CQRr7XqVs
15. https://www.youtube.com/watch?v=RwmHY-pp4m0
16. https://www.youtube.com/watch?v=SiC56gcP2bY
17. https://www.youtube.com/watch?v=iPowVuj8scU
18. https://www.youtube.com/watch?v=bhAoiKmHTQU
19. https://www.youtube.com/watch?v=UXwJ51ncmRQ
20. https://www.youtube.com/watch?v=D3SYfjZBjd4
21. https://www.youtube.com/watch?v=o89zHXNZ_pM (link no longer available at time of publication) 22. https://www.youtube.com/watch?v=zbXPf0JbUDA
23. https://www.youtube.com/watch?v=1vKlD3va35I
24. https://www.youtube.com/watch?v=S9F0F1qqMbA
25. https://www.youtube.com/watch?v=CGTfxGcX1Ok
26. https://www.youtube.com/watch?v=Q-2JxJB-3gU
27. https://www.youtube.com/watch?v=28BO6VlDgMs
28. https://www.youtube.com/watch?v=c9A_La_zhkc
29. https://www.youtube.com/watch?v=G-Y7aLouq0E
30. https://www.youtube.com/watch?v=N-REBe3FRIw
31. https://www.youtube.com/watch?v=Erw9Mn537V0
32. https://www.youtube.com/watch?v=7Ajwo9yRxOs
33. https://www.youtube.com/watch?v=HxU97lfbDlw
34. https://www.youtube.com/watch?v=nbM-5jk3YAk
35. https://www.youtube.com/watch?v=XrHJGhLN8e0
36. https://www.youtube.com/watch?v=rYMqXsAxzL8
37. https://www.youtube.com/watch?v=_9Mnd_rvDEY
38. https://www.youtube.com/watch?v=NTUmnZk2UW0
39. https://www.youtube.com/watch?v=QhktLyKzgxQ
40. https://www.youtube.com/watch?v=f-XpRn1siR4
41. https://www.youtube.com/watch?v=IGmgra_Ic6I
42. https://www.youtube.com/watch?v=Z_SsWTEcsFs
43. https://www.youtube.com/watch?v=AAz6o7S-eQU
44. https://www.youtube.com/watch?v=m5BVivcLiuQ
45. https://www.youtube.com/watch?v=mtlQ6ukDZCQ
46. https://www.youtube.com/watch?v=s6Pugtzqidc
47. https://www.youtube.com/watch?v=asxLVDlXVnE (link no longer available at time of publication) 48. https://www.youtube.com/watch?v=czeGS_p-yzU
49. https://www.youtube.com/watch?v=PZd8oxvXL6k (link no longer available at time of publication) 50. https://www.youtube.com/watch?v=cwiZGyo0PVg
51. https://www.youtube.com/watch?v=9eOW3B0NGDk
52. https://www.youtube.com/watch?v=htBz19FypHE
53. https://www.youtube.com/watch?v=duftSB9UP9s
